# Inhalation injury in critical burn patients

**DOI:** 10.1186/cc12056

**Published:** 2013-03-19

**Authors:** L Cachafeiro, M Sanchez, E Herrero, L Fernández, M Irazabal, M Hernandez, A Agrifoglio, A Garcia de Lorenzo, M Lendinez

**Affiliations:** 1 Hospital La Paz, Madrid, Spain

## Introduction

The objective is to analyze the epidemiology and mortality of critical burn patients with inhalation injury.

## Methods

A prospective, observational and descriptive study was carried out in all patients admitted to an ICU from October 2008 to June 2011. Inhalation injury was defined with two or more of the following criteria: history of injury in an enclosed space, facial burns with singed nasal hair, carbonaceus sputum or stridor. If they were intubated it was diagnosed by bronchoscopy. Demographic data, length of stay, ABSI, APACHE II, duration of mechanical ventilation, hospital course and mortality data were collected. Data are presented as number and percentage or as median and interquartile range and were analyzed with the Fisher exact test and Mann-Whitney test.

## Results

Of the 362 patients admitted, 84 (23.2%) had inhalation injury. Seventy-six percent were male and the average age was 52 (± 17.5) years. The mean total burn surface area (TBSA) was 28% (± 25). Forty-five patients (53.5%) had TBSA >20%. These patients had higher severity scores: ABSI 8 (± 2.8) versus 6 (± 2.2) (*P *= 0.0001), APACHE II 13 (± 6.9) versus 7 (± 6.7) (*P *= 0.0001). They also had a higher need for mechanical ventilation (88.1% vs. 34.9%, *P *= 0.001) and shock (59.5% vs. 27.0%, *P *= 0.001). The PaO_2_/FiO_2 _ratio at admission with and without inhalation was 245 versus 359 (*P *= 0.003), and at 72 hours was 207 versus 306 (*P *= 0.002). There were no significant differences in the appearance of sepsis, ARDS and renal failure. The length of stay with and without inhalation was 25.2 (1 to 95) days versus 22.5 (1 to 92) days (*P *= 0.4). The inhalation injury group showed a significantly higher mortality (9.0% vs. 28.9%, *P *= 0.001).

## Conclusion

In this study, inhalation injury is common in burn patients. They had higher severity scores (APACHE II, ABSI) and higher mortality. These patients had a higher need for mechanical ventilation and lower PaO_2_/FiO_2 _ratio, but there was no significant increase in ARDS or respiratory sepsis.

